# Balloon-guided inflation osteoplasty in the treatment of Hill-Sachs lesions of the humeral head: case report of a new technique

**DOI:** 10.1186/s13037-016-0092-y

**Published:** 2016-02-01

**Authors:** Gunther H. Sandmann, Sebastian Siebenlist, Florian B. Imhoff, Philipp Ahrens, Markus Neumaier, Thomas Freude, Peter Biberthaler

**Affiliations:** Department of Traumatology, Klinikum rechts der Isar, Technical University Munich, Ismaninger Str. 22, D-81675 Munich, Germany; Department of Traumatology, Eberhard-Karls-Universitaet, Schnarrenbergstr. 9, D-72076 Tuebingen, Germany; Department of Orthopaedic Surgery, Krankenhaus Barmherzige Brueder, Romanstr. 93, D-80639 Munich, Germany; Department of Orthopaedic Surgery, Klinikum Mittelbaden, Lilienmattstr. 5, D-76530 Baden-Baden, Germany; Department of Orthopaedic Sports Medicine, Technical University Munich, Ismaninger Str. 22, D- 81675 Munich, Germany

**Keywords:** Shoulder dislocation, Hill-Sachs lesion, Balloon osteoplasty

## Abstract

**Background:**

The use of the extra-vertebral balloon osteoplasty is increasing and in the meanwhile it has become a safe surgical technique in the treatment of tibial head, distal radius and calcaneus fractures. In addition, we already could show in a biomechanical setup that the balloon osteoplasty might be a safe tool for reduction in the treatment of Hill-Sachs lesions, but clinical application has not been performed so far.

**Case presentation:**

We report the case of a 53 year- old male patient who was referred to our Trauma department (level I trauma center) after the first manifestation of a posterior shoulder dislocation due to an epileptic seizure, originated in a- up to this date unknown -glioblastoma. After closed reduction of the dislocated shoulder the X-ray showed a subcapital fracture of the proximal humerus with a large reversed Hill-Sachs lesion. We performed an open surgery via a deltoideopectoral approach and balloon osteoplasty was used to reduce the impression fracture (Hill-Sachs lesion) before fixing the fracture with a locking plate. In the post-operative CT scan we could show an anatomical reduction of the Hill-Sachs lesion. At the follow-up examination one year after surgery the patient reached full range of motion and stated no re-dislocation of the shoulder nor instability or pain.

**Conclusion:**

The reduction of an impressed humeral head fracture by use of balloon osteoplasty is a safe technique. This technique could be a new option in the treatment of Hill-Sachs lesions and might be an alternative to well known standard procedures like the remplissage or tendon transfers without affecting rotation.

## Background

The use of the balloon-guided kyphoplasty is a safe and reliable operative technique [1] and in the meantime several extra-vertebral applications have been described. In this meaning reports about the balloon osteoplasty reduction concerning tibial head, the distal radius and calcaneal fractures [2–5] can be found. In addition we already could show in an experimental setup that the balloon- osteoplasty might be an alternative in the treatment of reverse Hill-Sachs lesions [6]. This entity is caused by the impaction of the humeral head on the posterior glenoid rim and is often the result of seizures [7] and often bilateral [8] and is associated with recurrent instability in up to 30 % [9]. In fact - apart from the inverted pear-shape glenoid- the engaging Hill-Sachs lesion is reported to be one of the reasons for failed shoulder stabilizations [10]. However, the treatment of reverse Hill-Sachs lesions is vague and diverse treatment options exist ranging from percutaneous correction [11], osteochondral allograft transplantations [12], rotational osteotomies [13] to remplissage maneuvres as proposed by Park et al. [14] or tendon transferring gap closing procedures as proposed by Hawkins [15] and McLaughlin [16]. For those lesions comprising more than 40 % of the humeral head arthroplasty is regarded as the best possible treatment option [17]. Most of the known techniques are demanding and result in a limitation of range of motion. This case report describes – to our knowledge- the first safe clinical application of the balloon osteoplasty for the reduction of a reverse Hill-Sachs lesion in a patient with posterior shoulder dislocation.

## Case presentation

**Fig. 1:**
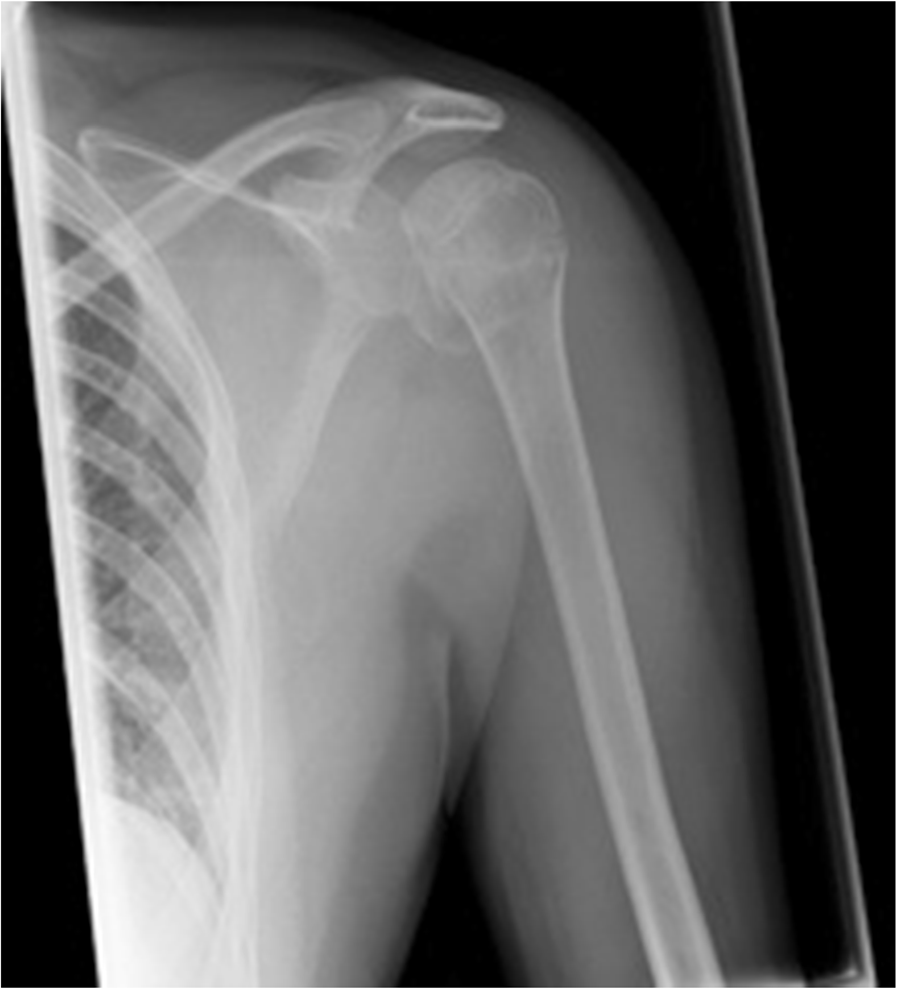
Pre-operative x-ray showing the posterior dislocation and the reverse Hill-Sachs lesion

A 53- year- old male patient who sustained a posterior shoulder dislocation with combined subcapital fracture and reversed Hill-Sachs lesion (see Fig. 1) was referred to our level I trauma center. The underlying cause was an epileptic seizure due to a recently diagnosed glioblastoma. Due to the morphology of the fracture and due to the size of the reverse Hill-Sachs lesion (27 % of the humeral head) a surgical intervention was recommended. In this special setup with restricted life expectancy the minimal-invasive technique thereby reducing operative trauma, allowing early mobilization and avoiding a lack of internal rotation was discussed with the patient. After informed consent, the operation was performed in a beach chair position via a deltoideo-pectoral standard approach.

**Fig. 2:**
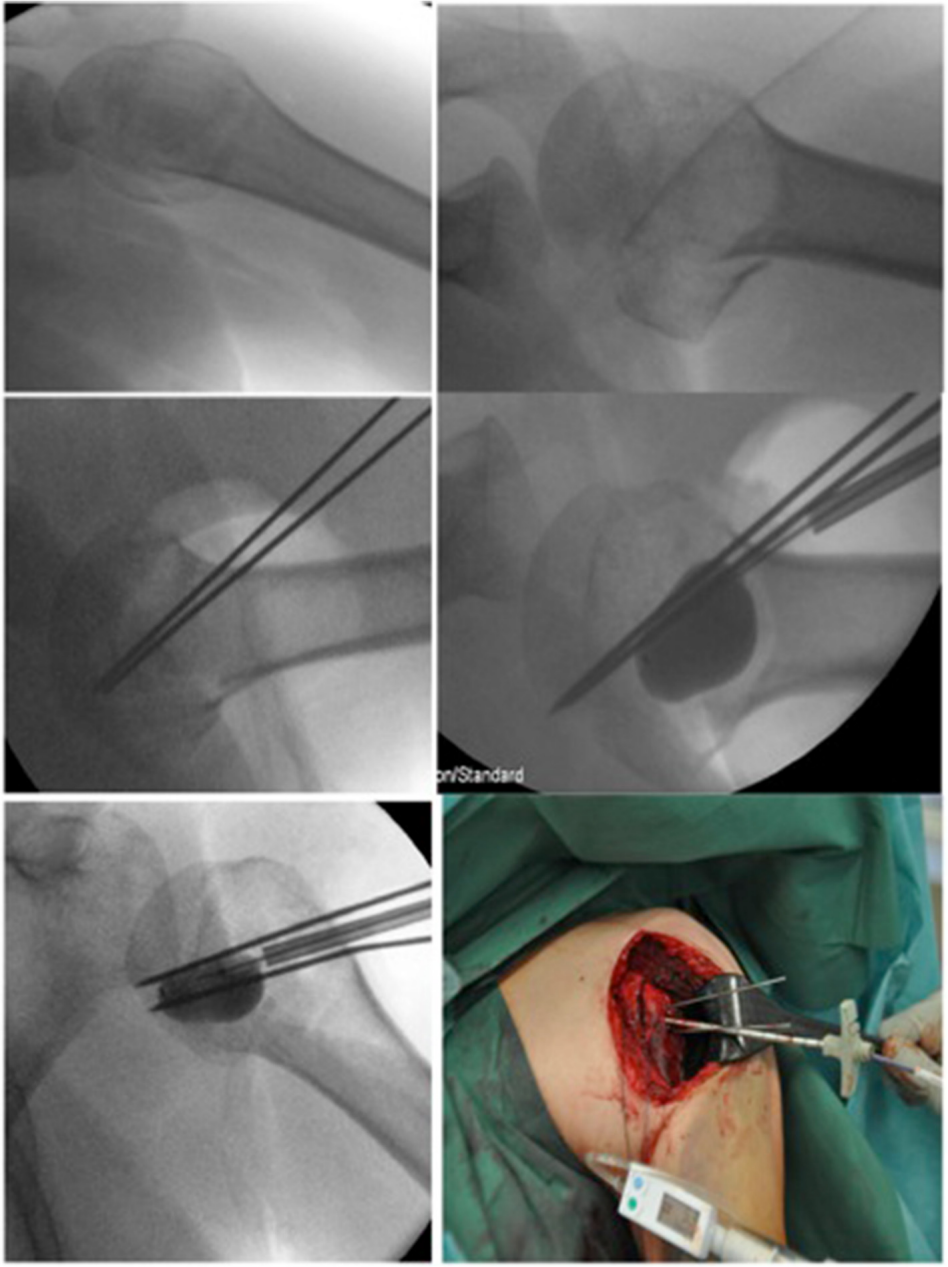
Intra-operative fluoroscopy and clinical image of the reverse Hill-Sachs lesion and the reduction maneuver using a kyphoplasty balloon. Note the K-wires used as counter-bearing/abutment

**Fig. 3:**
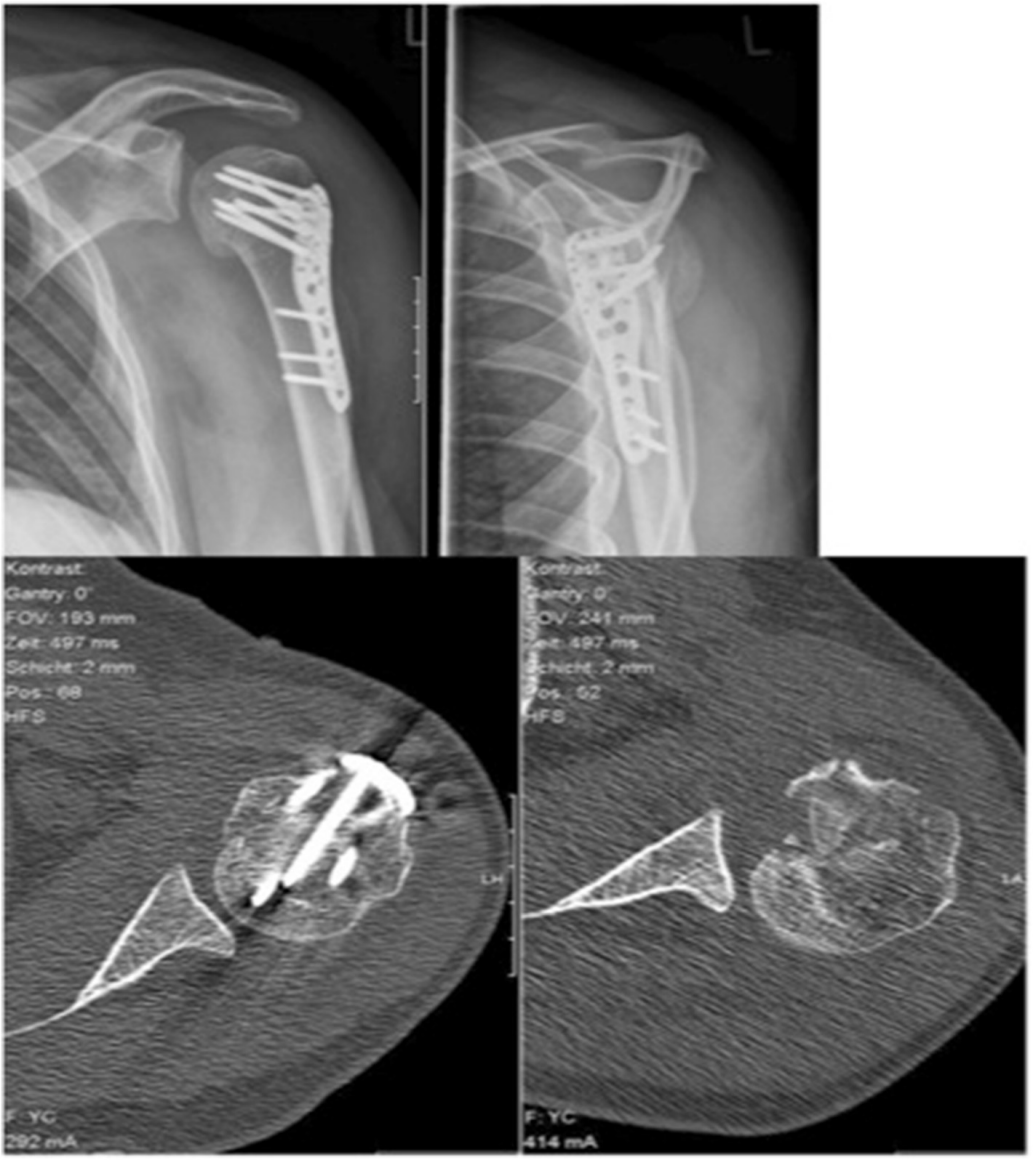
Post-operative x-ray and CT scan showing the restoration of spherical shape compared to pre-operative situation

In a first step the reverse Hill-Sachs lesion was reduced using an inflatable balloon as used in kyphoplasty. In contrast to our experimental set-up [6] were we had a fenestrated cannula as a stearing instrument, we used two 1.6 mm K-wires as a sort of counter bearing/ abutment [18]. The K-wires were inserted parallel to the impressed reverse Hill-Sachs lesion not perforating the contralateral cortical bone (see Fig. 2). As the “entry point” for the cannula we chose the major tubercle due to the good bone quality for reduction. The reduction maneuver was performed by using an inflatable balloon (Kyphon/Medtronic, Memphis, TN, USA; maximum of pressure of 100 PSI). To avoid pressure peaks we inflated the balloon several times in different positions and could thereby restore the spherical shape of the humeral head. As we have seen in our experimental set-up the balloon was positioned up to 3 mm to the depressed Hill-Sachs lesion. The reduction procedure was controlled under fluoroscopy and under visual control and lasted 15 minutes. After the successful reduction we stabilized and fixed the fracture by the use of a standard locking plate (Philos, Synthes Inc., Umkirch, Germany). Additional cerclages (Fiberwire No. 5; Arthrex Inc., Naples, USA)) for the minor and major tubercle were additionally applicated to control the reduction. By the position of the plate we were able to stabilize the augmented part of the Hill-Sachs lesion. In the post-operative X-ray and the CT scan a anatomic reduction of the impressed humeral head fracture was the result (see Fig. 3).

The patient recovered well from this surgical intervention, achieved a free range of motion and had no re-dislocation at the one year follow-up. No technique-related complications occured. Unfortunately, we are not able to report about the long-term follow-up as the patient died of complications caused by his glioblastoma 22 months after surgical intervention.

## Discussion

Posterior dislocation of the shoulder is a rare entity and therefore often misdiagnosed [19]. Once diagnosed, the therapy depends on the amount of the defect and the time from injury. Especially the size of the defect plays a crucial/ way guiding role in the further therapy. Defects up to 25 % and dislocations less than 3 weeks might be treated conservatively by closed reduction and immobilization in external rotation [20] whereas defects larger than 50 % of the articular surface should be treated with shoulder arthroplasty [17, 21]. The management of defects ranging from 25 to 50 % is still controversial and more challenging and debates on the best treatment options are ongoing. Mc Laughlin was the first surgeon who recognized the importance of impaction fractures in posterior shoulder dislocations. He recommended a subscapularis tendon transfer into the defect to avoid the engaging of the reverse Hill-Sachs lesion on the cost of compromised rotation. This technique was modified by Hawkins [15] recommending a transfer of the lesser tubercle and by Charalambous et al. [22] proposing a plication of the subscapularis tendon into the humeral head defect using suture anchors. All described techniques alter the humeral head anatomy, and compromise secondary prosthetic reconstruction and might lead to a limited internal rotation [23]. Therefore, several authors proposed more anatomic reconstructions using cancellous bone as autografts/ allografts for defect- filling of the impression fracture [12, 24, 25].

As an alternative technique Keppler et al. [13] described the treatment of locked posterior dislocations using a humeral rotational osteotomy. However, this method has not gained popularity due to technical difficulties and the risk of devascularisation of the humeral head. Hence, we were looking for a new technique of Hill- Sachs reconstruction with limited surgical trauma and immediate postoperative physiotherapy and no loss of internal rotation.

The presented case describes the reduction of the reverse Hill-Sachs lesion using a regular kyphoplasty balloon [6] in a first clinical application. We could achieve an anatomic reduction of the reverse Hill-Sachs lesion and the patient recovered without any complications as wound infection, failure of the osteosynthesis or re-dislocation. At follow-up 1 year after surgery, he had regained full range of motion and no shoulder-related problems during activities of daily living. Unfortunately, we are not able to report about the long-term result as the patient died of complications due to his glioblastoma 22 months after surgical intervention. However, especially in this situation it was important that the patient has a long time of remaining life quality with a limited surgical trauma. Hence we applied this new technology, since it combines good reduction with a very limited surgical trauma and allows the patient immediate movement and physiotherapy.

Some questions are left unanswered and remain for future research. Though we used a open deltoideo-pectoral approach due to the accompanying subcapital fracture and the need of additional plating we are convinced that this technique is suitable under arthroscopic conditions thereby further reducing the surgical trauma and remaining minimal-invasive. From our own experience the Hill-Sachs lesions has then to be treated as close to the accident as possible. To enable exact arthroscopic intervention a guiding instrument as we know it from ACL reconstruction might be helpful as well as a fenestrated cannula likewise we used in tibial head reconstruction as an abutment and lesion indicator [5]. In addition, the arthroscopic approach would be useful to address accompanying intra-articular comorbidities as labral lesions, pathologies of the biceps tendon or rotator cuff tears.

The other remaining point is the matter of defect filling after reduction of the reverse Hill-Sachs lesion. In our case we were able to save the reduction by positioning of the locking plate. In those cases where you would have an isolated impaction fracture it might be sufficient to limit range of motion to leave the defect unaffected for the first 6 weeks. As an alternative, defect filling would be possible either with cancellous bone or bone substitues and would probably be superior to filling of the emerging hollow with bone cement as described by Jacquot et al. [4] in the treatment of calcaneal fractures thinking of the generally younger age of our patients. In addition, the humeral head is a non-weight bearing bone and does not need the primary stability as desired in the treatment of calcaneal fractures.

Despite all positive reports on the inflation osteoplasty, this new surgical technique is not free from complications and as in all emerging techniques there is always the risk of potentially harm patients until the technique is well established and validated [26]. In this context, Mauffrey et al. [27] reported on a complication rate related to this new surgical technique of 65 % in the treatment of tibial plateau fractures ranging from minor complications like balloon burst with leakage of the contrast dye or accidental extrusion of calcium phosphate to the posterior soft tissue to major complications consisting of intraarticular extrusion of calcium phosphate or the unability to elevate the depressed fragment by inflation osteoplasty.

However, this is a first report on the described technique in the reduction of a reversed Hill-Sachs lesion and further studies are needed with special remark on complications and their management.

## Conclusion

In our described case the reduction of the reverse Hill-Sachs lesion by use of inflation osteoplasty enabled us to achieve an almost anatomical reduction in the humeral head without limitation of external rotation as commonly found in patients after remplissage or tendon transfers and there we see the great advantage of the presented technique. In cases with isolated reverse Hill-Sachs lesions the application of the balloon osteoplasty might be even performed arthroscopically and thereby further reducing the comorbidity of the open surgical approach.

## Consent

Written informed consent has been obtained from the patient for the publication of this case report and any accompanying images.

A copy of the written consent is available for review for the editor-in-chief of this journal.
